# Supplementary material for: In-depth characterization and comparison of the N-glycosylated proteome of two-dimensional- and three-dimensional-cultured breast cancer cells and xenografted tumors
Source: PLoS One. 2020 Dec 10;15(12):e0243789. doi: 10.1371/journal.pone.0243789 (PMC7728280; doi:10.1371/journal.pone.0243789)
Supplement: S1 Fig — (DOCX) [file pone.0243789.s001.docx]

**Supporting information for**

**In-depth Characterization and Comparison of the N-glycosylated Proteome of Two-dimensional- and Three-dimensional-cultured Breast Cancer Cells and Xenografted Tumors**

Yonghong Mao^1,2,3^, Yang Zhao^4^, Yong Zhang^5^, Hao Yang^5*^

^1^Department of Thoracic Surgery, West China Hospital, Sichuan University, Chengdu 610041, China.

^2^Chest Oncology Institute, West China Hospital, Sichuan University, Chengdu 610041, China. ^3^Western China Collaborative Innovation Center for Early Diagnosis and Multidisciplinary Therapy of Lung Cancer, Sichuan University, Chengdu 610041, China.

^4^Mass Spectrometry Engineering Technology Research Center, Center for Advanced Measurement Science, National Institute of Metrology, Beijing 102206, China

^5^Key Lab of Transplant Engineering and Immunology, MOH; Institutes for Systems Genetics; Frontiers Science Center for Disease-related Molecular Network, West China Hospital, Sichuan University, Chengdu 610041, China.

*Corresponding author

E-mail: yanghao@scu.edu.cn (HY)

**S1 Fig.** The overlap of intact N-glycopeptides, N-glycoprotein, N-glycan compositions between two biological replicates of 2D (A) and 3D (B) cultured breast cancer cells *in vitro* and xenografted tumors (C) in mice *in vivo*.

**
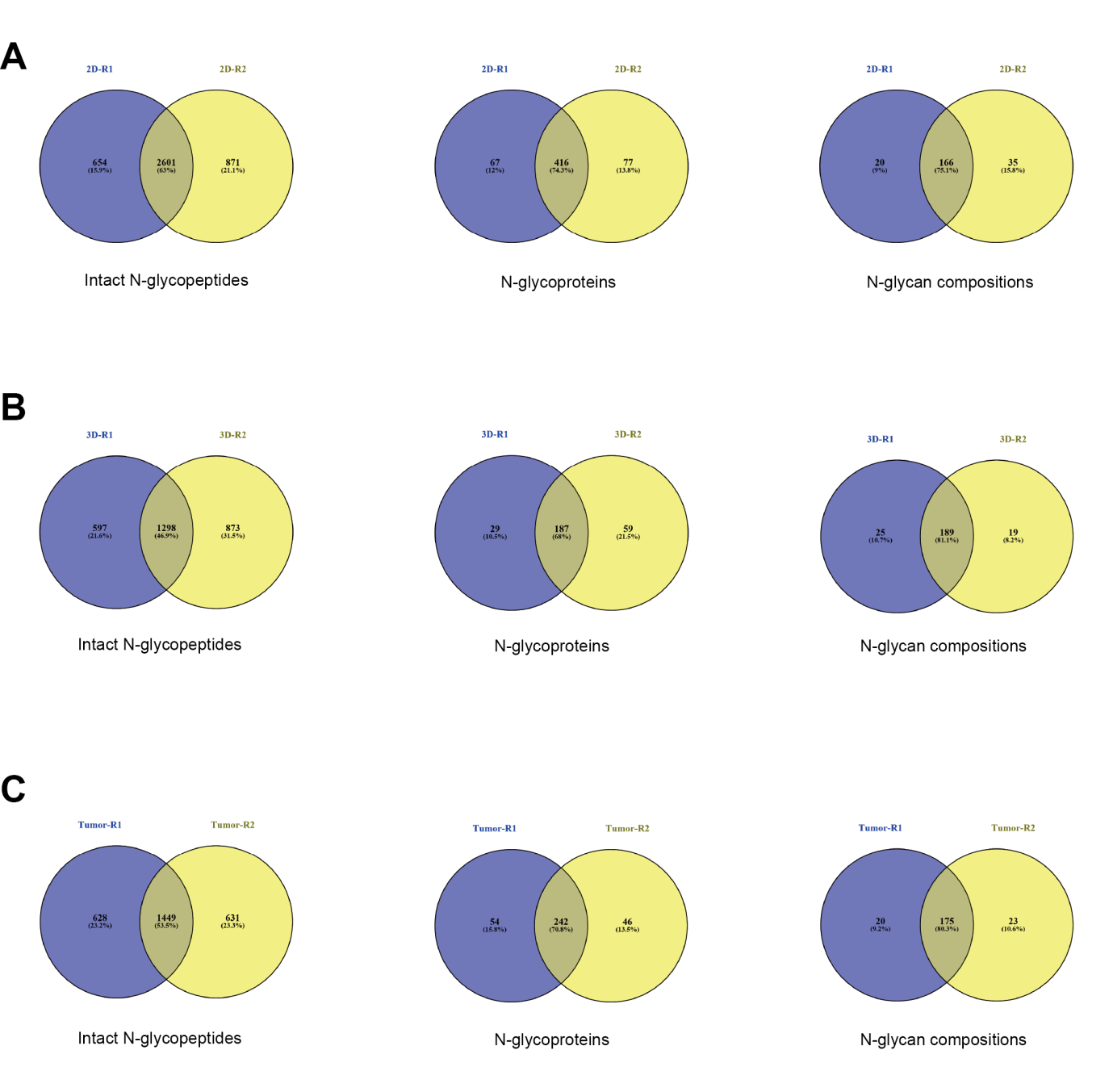
**
